# Shape Control of a Unimorph Deformable Mirror for Space Active Optics under Uncertainties

**DOI:** 10.3390/mi14091756

**Published:** 2023-09-09

**Authors:** Kainan Wang, Yian Yu, André Preumont

**Affiliations:** 1 School of Remote Sensing and Information Engineering, Hubei Luojia Laboratory, Wuhan University, Wuhan 430072, China; yuyian@whu.edu.cn; 2 Department of Control Engineering and System Analysis, Université Libre de Bruxelles (ULB), CP. 165-55, 50 Av. F.D. Roosevelt, B-1050 Brussels, Belgium; andre.preumont@ulb.be

**Keywords:** active optics, unimorph deformable mirror, space application, structural and environmental uncertainties

## Abstract

This paper focuses on the change of morphing capabilities for a unimorph deformable mirror impacted by environmental factors, which works in space for active optics applications. Various aspects of disturbing sources are considered, including complex thermal and mechanical conditions on ferroelectric behaviours of strain actuation, and influences of preconfigured initial shapes and stress-induced geometric stiffness on the structural rigidity of the mirror; changes on both the perturbed shape and the Jacobian matrix are discussed. Those variations are regarded as uncertainties in the design of control methods with both open-loop and iterative control strategies tested in the quasi-static range.

## 1. Introduction

The lightweight design of space observation satellites offers several advantages, including reduced launch and operational costs and the ability to respond quickly to space missions. However, achieving high-precision observation of certain celestial objects presents optical design challenges. In particular, large-aperture instruments are often required to improve the resolution of the observation system, necessitating a substantial set of telescope optics [1]. Moreover, lightweight space structures of considerable size exhibit significant compliance and can be easily perturbed by environmental excitations of various bandwidths. To address these conflicting design requirements, several technical solutions can be considered for the development of telescope optics for space observation. These solutions encompass optimized material choices for satellite structures to strike a balance between overall inertia and rigidity [2], intricate designs of retractable mechanisms to minimize volume during launch [3], and the utilization of lightweight telescope primaries to reduce areal densities [4]. Given the high flexibility of lightweight structures, critical on-board components are sensitive to the complex space environment, which can lead to changes in optical surfaces and misalignment of telescope optics layouts. Such alterations may result in optical wavefront distortion when feeding into on-board receivers, ultimately impairing imaging capabilities. Therefore, it is imperative to develop effective strategies to mitigate optical errors in space observation platforms.

**Figure 1:**
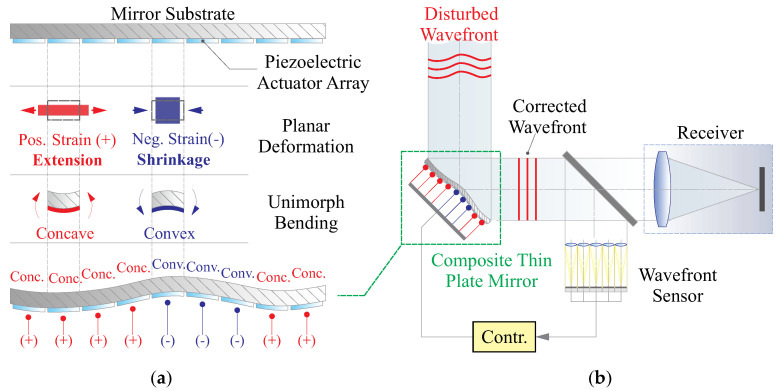
(**a**) Illustration of working principle of a unimorph deformable mirror with patterned strain actuators; (**b**) applications in a real-time wavefront correction system of a deformable mirror.

In contrast to conventional passive opto-mechanical designs for telescope optics, active real-time wavefront error correction may offer a more promising approach for future space telescopes. This approach involves the use of optical actuators to actively adjust the wavefront within a clear pupil. Examples of such actuators include Spatial Light Modulators (SLMs), lenses with variable refractive indices, deformable mirrors/reflectors, and metasurfaces. Among these options, deformable mirrors are the most commonly used in telescope optics, as they actively control the wavefront by changing their shape with an array of mechanical actuators on the mirror’s backside. For large ground-based telescopes, deformable mirrors serve as phase modulators, conjugated to atmospheric turbulence screens at specific altitudes, as part of adaptive optics (AO) systems. Other wavefront error correction techniques may occur at the telescope’s entrance pupil or collaboratively reduce phase deviations introduced by other components in the optical chain, often referred to as Active Optics (AO) [5,6]. Deformable mirrors can be categorized based on the direction of actuation forces. The first category employs force actuators or displacement actuators driven by out-of-plane (normal) forces. These actuators can consist of voice coils or piezoelectric stackers, which generate significant driving forces [7,8]. Mirrors equipped with these actuators typically require robust supporting structures to withstand reaction forces and are commonly used in large ground-based telescopes with sophisticated temperature and humidity control systems [9]. The second category of deformable mirrors uses strain actuators controlled by in-plane (tangential) forces, typically constructed from composite layered materials such as piezoelectric ceramics, e.g., Lead Zirconate Titanate (PZT) or polymers like PolyVinylidene Fluoride-co-TriFluoroEthylene (PVDF-TrFE) [10,11,12,13]. These transverse actuators generate self-equilibrium bending moments and forces that act on discrete areas to achieve a desired shape. Unimorph active mirrors have been employed in early-stage ground telescopes and have recently garnered attention from aerospace communities due to their lightweight, compact design, and low power consumption, although sensitive to environmental factors, those unique advantages still make them suitable for space applications, attracting sufficient attention from the aerospace community [14]. Figure 1 illustrates the working principle of a unimorph deformable mirror with strain actuators and its application in a real-time wavefront correction system.

In comparison to ground-based telescopes, space telescopes face a more complex operating environment due to the special challenges posed by working in space. Satellites in space are subject to variations in thermal and gravitational conditions, leading to structural deformations that can result in misalignment of optical surfaces and the introduction of wavefront errors, often in the form of primary aberrations. To mitigate these errors, deformable mirrors are employed to partially correct wavefront distortions, thereby improving the Strehl Ratio (SR) within the designated Field-of-View (FoV) [4]. However, it is important to note that the deformable mirror itself is susceptible to environmental changes, which can lead to distortions in its own pupil. These distortions may arise from various factors, such as thermomechanical coupling or the release of residual stresses from composite layers, among others [15]. The use of a unimorph mirror in space presents additional challenges, as it is sensitive to environmental factors that affect its morphing behaviour. This paper specifically focuses on the example of the unimorph piezoelectric actuator, whose stroke can be significantly influenced by large temperature variations [16,17]. Other factors that can alter the actuator’s morphing capabilities include the magnitude of planar stress applied to the active film and the substantial bias field required to ensure positive polarization [18,19]. Furthermore, the morphing behaviour of the active mirror can be influenced by its stress status within the mirror substrate. Non-uniform edge loads from the mounting boundaries or residual stresses within multiple layers can introduce nonlinear geometric stiffness, altering the overall rigidity of the structure [20]. In addition to these factors, the performance of wavefront control in a space active optics system is impacted by the working status of the actuators. For example, piezoelectric materials may degrade or depolarize over time, leading to a reduction (or even failure) in strain generation by the active layer.

This paper emphasizes shape control strategies for unimorph deformable mirrors in the presence of structural uncertainties due to environmental factors. The study places particular emphasis on the potential utilization of active mirrors in space, and it comprehensively investigates various decoupled factors, a topic that has been rarely explored to our knowledge. The study relies extensively on numerical investigations using Finite Element (FE) analysis for mechanics and mechatronics analysis. This paper is organized as follows: Section 2 gives a short overview of the framework of the quasi-static control for forming a shape, starting with a mathematical formulation on the interaction matrix and the Least Squares (LS) techniques are discussed. Section 3 conducts detailed mechanics analysis on the morphing behaviour of a deformable mirror numerically under certain scenarios, and changes on both disturbed shape and the Jacobian matrix are discussed. The first scenario considers a complex thermal/mechanical condition on the ferroelectric behaviour of the active film, and then the effects on the morphing strokes, variated by the initial shape and stress-induced geometric stiffness of the mirror substrate, are analysed. Section 4 proposes a method of analysing the perturbed Jacobian matrix by the Singular Value Decomposition (SVD) with uncertainties, finally the strategies of open-loop reconstruction and iterative control based on the Damped Least Squares method are studied, with various environmental factors applied for the numerical verification.

## 2. Framework of Quasi-Static Control

This section gives a review of the framework for the quasi-static control of the shape of a piezoelectric deformable mirror [21], which is unimorphly controlled by a set of strain actuators, the driving voltages are fed to the patterned electrodes with a certain shape formed. The structural dynamic equation with respect to a discrete Degree-of-Freedom (DoF) q of the mirror surface can be written by

(1) $$M\ddot{q}+C\dot{q}+Kq=K_{a}v$$

where *M*, *C*, and *K* are the inertial, damping, and stiffness matrices of the mirror structure, respectively, $K_{a}v$ is the morphing forces driven by unimorph strain actuators, in which v is the vector of actuator inputs (voltages), and $K_{a}$ stands for the matrix relating the input voltages to the equivalent piezoelectric forces acting on the mirror. The structural dynamics of the active mirror are usually ignored in case of a low bandwidth shape correction for the application of space optics, i.e., $\ddot{q}=\dot{q}=0$, the shape control can be considered *quasi-static*. Other techniques for the dynamic control of a deformable mirror can be found in [22].

In the presence of a wavefront sensor measuring the shape of the mirror with an output of **s**, the system input–output relationship is

(2) $$s=C_{S}q=C_{S}K^{-1}K_{a}v=Jv$$

where $C_{S}$ is the pupil function defining the usable part of the full mirror surface in the optical system. There are several methods for monitoring wavefront shapes: (1) decoding varying fringes interfered with a reference coherent light beam (based on diffractive optics), as seen in references such as [23,24,25], this method is commonly employed in metrological systems for surface examination, offering ultra-high measurement accuracy (λ/1000) but with a relatively slow update rate; (2) reconstructing spot locations focused on the sensor panel with a micro-lens array (based on geometrical optics), as discussed in works like [26,27], this approach is based on the well-known Shack–Hartmann configuration [28], providing real-time wavefront sensing with the use of a Guide Star (GS); (3) retrieving phase information by comparing intensities at image and focal planes without specific sensors, as demonstrated in [29,30]. In this paper, we assume perfect shape monitoring without considering sensor dynamics and noise. Our focus is primarily on the optical actuator. However, it is important to note that in practical applications, even in an iterative feedback loop, the sensor error (noise) should be controlled to significantly lower level than wavefront errors produced by the mirror surfaces in telescope optics. Achieving this low sensor noise is a challenging aspect of implementing AO in space. We define the mapping matrix $J=CK^{-1}K_{a}$ (called *Jacobian*), associating the sensor output s to the voltage input v. Note that Equation (2) is commonly overdetermined, and thus the inverse problem can be solved in the sense of Least Squares (LS)

(3) $$v=J^{+}s_{t}$$

where $J^{+}={\left(J^{T}J\right)}^{-1}J^{T}$ is the pseudo inverse of *J*. The vector $s_{t}$ is the target shape to be formed, and this shape is designed to be compatible with other surfaces in telescope optics, enabling flexible deployment of the optical layout and correcting wavefront errors caused by various sources. The forming error of a target shape is quantified by the Root Mean Square (RMS) of the error shape, i.e., $∥\left(I-JJ^{+}\right)s_{t}{∥}_{2}$ for Equation (3). Other methods of inverting *J* can be *Tikhonov regularization* with the voltage solved by

(4) $$v={\left(J^{T}J+\alpha^{2}I\right)}^{-1}J^{T}s_{t}$$

where $\alpha^{2}$ is the weighting factor balancing the forming error $\left|\right|s_{t}-Jv{\left|\right|}_{2}$ and the voltage consumption ${\left||v|\right|}_{2}$ in the minimization problem of

(5) $$\min_{v}\left(|\right|s_{t}-{Jv||}_{2}^{2}+\alpha^{2}{\left||v|\right|}_{2}^{2})$$

the method is also known as Damped Least Squares (DLS) [31].

In addition to forming a target shape, the deformable mirror should also be actively controlled to compensate in real time the deviated shape (the part within the pupil is denoted by $s_{d}$) disturbed by environmental or structural factors, e.g., excitations by thermal sources or variation of stress status. In general, compensating $s_{d}$ and forming $s_{t}$ can be treated separately, and reconstructed by $v_{d}$ and $v_{t}$, respectively, with certain bandwidths.

## 3. Mechanics of Morphing Behaviour of a Deformable Mirror with Environmental and Structural Uncertainties

The active optics units (based on piezoelectric materials) working in space suffer from a harsh thermal environment and complex stress condition, which poses influences on both the deviated shape $s_{d}$ and the Jacobian *J*. First, the time-varying deviation $s_{d}$ is commonly caused by non-uniformity of the thermal expansions for various materials, e.g., composite layers of attaching the mirror substrate with piezoelectric ceramics and boundary connections between the deformable mirror and supporting frames, those thermal sources might be acting externally, e.g., the radiation by celestial bodies and heat transfer from supporting frames, or internally, e.g., intrinsic thermal loads by the pyroelectric coupling, especially when a large electric field is applied to the thin piezoelectric film. The stress status is another source causing unwanted deformation of the mirror by a uncontrollable release process, this might come from an inappropriate manufacturing with residual stress under on-Earth gravitational environment or mechanical incompatibility with adherent structures.

Second, the Jacobian *J* of the deformable mirror is also impacted by those environmental factors and structural uncertainties, e.g., the temperature variation with a large range can affect the dielectric behaviour of the piezoelectric film, making a change on the value of the piezoelectric coefficient. On the other hand, the residual stress within those mirror layers slightly modifies the structural rigidity by introducing geometric stiffness, leading to a nonuniform change on the morphing strokes for strain actuators. A general theoretical formulation on the varied Jacobian can be revised from Equation (2) and expressed as

(6) $$J+\Delta J=\kappa C_{S}{\left(K+\Delta K\right)}^{-1}\left(K_{a}+\Delta K_{a}\right)B$$

where κ is the amplification factor for the piezoelectricity, impacted mainly by the temperature; the variation of stiffness ΔK (geometric stiffness) is made by the surface residual stress and tension/compression by the installing boundaries (mechanical incompatibility); $\Delta K_{a}$ represents changes on the magnitudes and directions of equivalent piezoelectric forces acting on the mirror, especially when formed or initial curvatures exist. Finally, the matrix *B* presents the status of each actuator (e.g., thermal/mechanical depoling or ageing effect on piezoelectric films, input noises or malfunctioning of the voltage amplifier, etc.), this matrix is considered to be diagonal, without the crosstalk between neighbouring actuators.

In this section, we conduct a detailed mechanics analysis on the morphing behaviour of a deformable mirror numerically under certain scenarios, changes on both $s_{d}$ and *J* are discussed. Note that sensing $s_{d}$ and *J* might be different; the deviated shape $s_{d}$ can be measured by the wavefront sensor and thus it can be compensated using the open-loop control strategies with the inversion of the Jacobian; however, the variation of *J* is not known by a direct sensing technique, and a iterative loop might be required for the reconstruction. Following structural parameters are used in the section: the thickness of the mirror substrate (made of silicon) and the morphing film (made of the PZT ceramics) are t=400 μm and $t_{p}=100$ μm, respectively; the deformable mirror has a diameter of $D_{M}=100$ mm with a clear pupil of $D_{P}=60$ mm, and is simply supported on the edge. The numerical model in this study is constructed using a linear FE program. It is based on the assumption that mechanical responses from various perturbing sources can be considered additive. This linear framework can be extended to account for stress-induced stiffness by simply adding a geometric stiffness that is computed non-iteratively. Furthermore, the nonlinearities in the behaviour of the load and material properties are treated as quasi-static, which results in constant estimates for these factors over specific time intervals.

### 3.1. Ferroelectric Behaviour of Actuation Layer

We can start with an assessment on the amplification factor (κ in Equation (6)) for the piezoelectric layer, or more specifically, ferroelectric materials under certain thermal/mechanical conditions. The ferroelectricity means a spontaneous polarization in addition to the piezoelectricity, commonly used piezoelectric materials (e.g., PZT, PMN-PT and PVDF) are ferroelectric.

A general analysis is given firstly on the thermodynamic functions for ferroelectric materials. The internal energy, *U*, describing the overall energies of various kinds within a system, can be expressed by the functions of the entropy *S*, the strain *x*, and the electric displacement *D*; a total derivative of *U* is

(7) dU=TdS+Xdx+EdD

where the conjugated variables *T*, *X*, and *E* are the temperature, the stress, and the electric field, respectively. There are several representations of potentials (free energies) of a thermodynamic system [16], with Legendre transformations of *U*; in this paper, particularly, we focus on the elastic Gibbs free energy $G_{E}=U-TS-Xx$, whose form is convenient for analysing the nonlinear behaviours of ferroelectrics [32]. The total derivative of $G_{E}$ is

(8) $$dG_{E}=-SdT-xdX+EdD$$

indicating that $G_{E}$ can be written as a function of *T*, *X*, and *D*, note that, the electric displacement *D* is commonly replaced by the polarization *P* (i.e., D≈P) for those materials with a high dielectric constant. In this sense, the Taylor series of the variation of $G_{E}\left(P,X,T\right)$ expanded at a certain state $G_{E,0}=G_{E}\left(P_{0},X_{0},T_{0}\right)$ can be written as

(9) $$G_{E}\left(P,X,T\right)=G_{E,0}+\sum a_{ijk}{\left(P-P_{0}\right)}^{i}{\left(X-X_{0}\right)}^{j}{\left(T-T_{0}\right)}^{k}$$

where $a_{ijk}$ are scaled partial derivatives (coefficients) of terms in the polynomial for different orders. According to Landau and Devonshire’s phenomenological theory for the phase transformation and electromechanical coupling [33], some of those coefficients in Equation (9) should be reserved and considered to be almost constants. In this study, we consider a ferroelectric film polarized along the direction of the thickness (denoted by 3), which performs uniaxial in-the-plane expansion (denoted by 1).

Equation (9) can be simplified for the case of the reference free energy shifted to $G_{E,0}=0$, this is usually defined at the origin for a unpolarized ($P_{0}=0$) state with a free mechanical boundary condition ($X_{0}=0$), an approximate of the variation of $G_{E}$ under an isothermal process can be written as

(10) $$G_{E}=\left(\frac{1}{2}b_{0}P_{3}^{2}+\frac{1}{4}b_{1}P_{3}^{4}+\frac{1}{6}b_{2}P_{3}^{6}\right)-\frac{1}{2}s_{11}X_{1}^{2}-Q_{31}X_{1}P_{3}^{2}-\alpha_{T}X_{1}\left(T-T_{0}\right)$$

where the first part (the polynomial related to *P*) represents the electric energy, $b_{0}∼b_{2}$ are Landau parameters and $b_{0}$ is temperature dependent $b_{0}=\left(T-T_{C}\right)/\varepsilon_{0}C_{T}$, in which $\varepsilon_{0}=8.854\times 10^{-12}$ F/m is the vacuum permittivity, and $C_{T}$ is *Curie–Weiss* constant. The phase transition temperature $T_{C}$ is usually equal to or slightly lower than the *Curie* temperature of the ferroelectric material, and above this temperature, the piezoelectric actuation cannot be achieved due to the paraelectric phase transition. The remaining parts of Equation (10) are the mechanical counterpart $-s_{11}X_{1}^{2}/2$ in the free energy $G_{E}$ where $s_{11}$ is the membrane compliance (for uniaxial loads), the electromechanical coupling term $-Q_{31}X_{1}P_{3}^{2}$ where $Q_{31}$ is the transverse electrostrictive constant, and the thermo-mechanical coupling term $-\alpha_{T}X_{1}\left(T-T_{0}\right)$, $\alpha_{T}$ is the Coefficient of Thermal Expansion (CTE). The nonlinear constitutive equations of a ferroelectric film can be obtained by computing the partial derivatives of $G_{E}$, which combines Equations (8) and (10),

(11) $$\begin{array}{cc} E_{3} & =\frac{\partial G_{E}}{\partial P_{3}}=\left(b_{0}-2Q_{31}X_{1}\right)P_{3}+b_{1}P_{3}^{3}+b_{3}P_{3}^{5}\\ x_{1} & =-\frac{\partial G_{E}}{\partial X_{1}}=s_{11}X_{1}+Q_{31}P_{3}^{2}+\alpha_{T}\left(T-T_{0}\right) \end{array}$$

The first equation describes the nonlinear dielectric behaviour, in particular, if there is no external bias field $E_{3}=0$, the solution $P_{3}=P_{S}$ is the spontaneous polarization, which is a measure of the saturation for the poling process of the ferroelectric film. The second equation of Equation (11) computes the strain of the film, and the nominal transverse piezoelectric constant can be defined by $d_{31}=\partial x_{1}/\partial E_{3}$, which defines the microscale strain with respect to the electric field in a linear sense.

**Figure 2:**
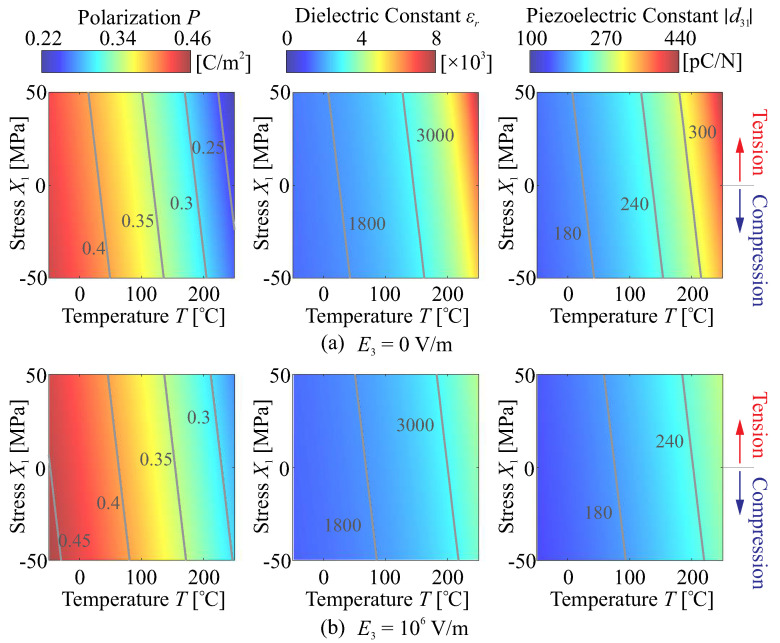
Computed results of an example ferroelectric material (thin layer) by solving Equation (11), the following material data are used: $T_{C}=350\;{}^{\circ}C$, $Q_{31}$ = −0.014 m${}^{4}/C^{2}$, $C_{T}=1.42\times 10^{6}$ K, with calibrated data of $d_{31}$ = −180 pC/N, and $\varepsilon_{r}=1800$ at the reference point ($X_{1,0}=0$ MPa, $E_{3,0}=\;0$ V/m, and room temperature $T_{0}=25\;{}^{\circ}C$).

Figure 2 gives the computed solutions of Equation (11) for an example of the piezoelectric ceramics, three parameters are examined: the spontaneous (internal) polarization $P_{S}$, the relative dielectric constant $\varepsilon_{r}$, and the transverse piezoelectric coefficient $d_{31}$ (magnitude) within a ferroelectric state, the environmental factors consider a wide range of temperature (below the phase transition temperature $T_{C}$) and stress variation, and also the effect of the large bias field is analysed. According to this, below $T_{C}$, the internal polarization $P_{S}$ is a decreasing function of the temperature, while $\varepsilon_{r}$ and $|d_{31}|$ increase with the temperature. Note that if the temperature increases close to the phase transition point $T_{C}$, the dielectric capacity and piezoelectricity will sharply reduce to zero, this is a unwanted scenario for the piezoelectric actuation in space with a complex thermal environment. The tension stress state and the low bias field are also augmenting the piezoelectricity of the unimorph layer.

Note that the free energy model of Equation (10) is proposed phenomenologically, and the microscopic uncertainties still remain. In practice, the environmental tests are required for a more precise calibration of the variation on the morphing capability of the piezoelectric film. In this paper, it is not intended to substitute those experiments by a theoretical model, and the order of magnitude of the bounded functions on ferroelectric behaviours affected environmental factors are focused; a literature survey shows a quantitative consistency of experimental results with respect to Figure 2 of various samples of piezoelectric ceramics (e.g., [17,18,19,34,35,36]), especially for hard PZT materials.

#### Thermal Deformation

The space structures are exposed to a complex thermal environment in space, which usually experience the temperature variation of a large range with a deteriorated condition of thermal transfer, and examples of thermal design on the space structures can be found in [37]. The thermal deformation would dominate the wavefront error of the optical surface, and for a unimorph piezoelectric deformable mirror, this is mainly caused by the mismatch of the CTE between the mirror substrate and the active film, and the change of the poling strain on the ferroelectric layer. In this study, we consider a uniform temperature change applied on the mirror structure, and the numerical sturdy on the thermal deformation for the large scale thin shell mirror can be found in [38].

We can return to the nonlinear dielectric behaviour in Equation (11), and rewrite the inverse function of the electric field along the thickness $E_{3}$:

(12) $$P_{3}=P_{S}+c_{i}E_{3}^{i}$$

where $c_{i}$ is the coefficients of the dielectric permittivities for the order *i*, e.g., $c_{1}=\varepsilon_{r}\varepsilon_{0}$. Assuming a stress-free condition, we can substitute Equation (12) into Equation (11) and obtain

(13) $$x_{1}=Q_{31}{\left(P_{S}+c_{i}E_{3}^{i}\right)}^{2}+\alpha_{T}\left(T-T_{0}\right)\approx Q_{31}P_{S}^{2}+d_{31}E_{3}+\alpha_{T}\left(T-T_{0}\right)$$

in which $Q_{31}P_{S}^{2}$ is the poling strain and $d_{31}E_{3}$ is the field-driven strain, it can be piezoelectric or electrostrictive (depending on the polynomial order of $E_{3}$ in the expression of $d_{31}$), a simple expression with no bias field can be $d_{31}{|}_{E_{3}=0}=2\varepsilon_{r}\varepsilon_{0}Q_{31}P_{S}$ (see [16,32]), various experimental procedures of calibrating those parameters can be found in [12].

Rough estimates on those strains of aberrated sources can be made under a temperature change of ΔT, the variation of the poling strain $x_{P}=Q_{31}\left[{\left(P_{S}+\Delta P_{S}\right)}^{2}-P_{S}^{2}\right]$ and the mismatch thermal strain $x_{T}=\Delta \alpha_{T}\Delta T$ are computed using following data: $Q_{31}=-0.014$ $m^{4}/C^{2}$, $\Delta \alpha_{T}=1.41\times 10^{-6}$ where $\alpha_{T,S}=4\times 10^{-6}$ for the mirror substrate (Silicon) and $\alpha_{T,P}=2.59\times 10^{-6}$ for the active layer (PZT). Consider a temperature increase ΔT=50°C from the reference temperature of $T_{0}=25\;{}^{\circ}C$, according to Figure 2, the spontaneous polarization decreases from the original value $P_{S}=0.403\;C/m^{2}$ to $P_{S}+\Delta P_{S}=0.376\;C/m^{2}$, where the change of the poling strain is $x_{P}=2.96\times 10^{-4}$, and the thermal strain is $x_{T}=7.05\times 10^{-5}$. On the other hand, we assume that the piezoelectric strain $x_{E}=d_{31}E_{3}$ of active control is made by applying unit voltages on an active layer of 100μm thick, resulting in $x_{E}=-1.8\times 10^{-6}$ if the value of $d_{31}=-180\;\mathrm{pC}/N$ is used. Based on those results, the strains caused by the change of thermal factors can be more than two orders of magnitude larger than the morphing strain controlled by the unit voltage, indicating that a large voltage consumption might be required with little voltage budget for controlling other shape error. In addition, special measures should be considered for a proper thermal design, e.g., a good thermal management ensuring a small range of temperature variation [39,40,41], or an elaborate thermo-mechanical design of the composite deformable mirror with a thermal balancing configuration [11,15,22].

### 3.2. Variation on Morphing Capability with Surface Curvature

The variation in the morphing capability of a deformable mirror also occurs with the strain actuation with surface curvature, due to a slight modification in magnitudes and directions of piezoelectric forces, i.e., resulting in a change of $\Delta K_{a}$ in Equation (6). Note that the curvature can be made artificially as conic surfaces in the telescope optics, the techniques of moulding a thin-shell layer of a prescribed curvature are in development for the electronics industry [42,43]. At the same time, the surface curvature can be also uncontrollably generated due to manufacturing or release of residual stress, leading to a random initial shape, this is one of sources of deviated shape $s_{d}$.

**Figure 3:**
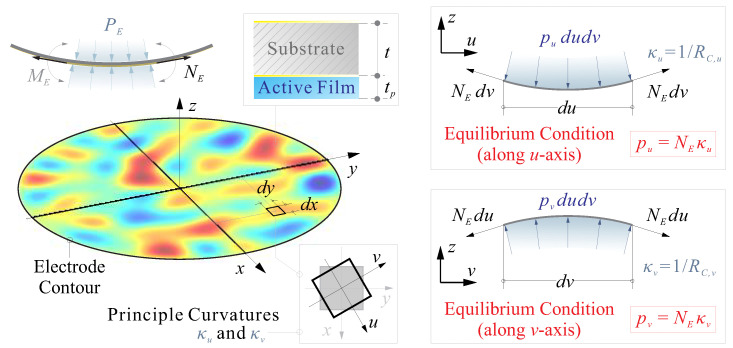
Illustrative diagram of equivalent loads for a surface with arbitrary curvatures, the mean curvatures are used to describe the profile of the mirror surface, the equivalent pressure balancing the edge force is analysed along orthogonal axes *u* and *v* defined in the sense of Gaussian curvatures.

The discussion might start with the unimorph strain actuation on a flat plate, which can be modelled by a set of equivalent forces consisting of a force normal to the contour of the electrode, which acts in the tangent plane, and a moment acting on the contour of the electrode. For voltage-driven (denoted by v) isotropic piezoelectric film actuators, the force and moment per unit length are, respectively [44],

(14) $$N_{E}=e_{31}v,\;M_{E}=e_{31}vt/2$$

where $e_{31}=d_{31}E_{p}/\left(1-\nu_{p}\right)$, $d_{31}$ is the transverse piezoelectric constant, and $E_{p}$ and $\nu_{p}$ are the Young modulus and the Poisson’s ratio of the piezoelectric material. For a spherical shell (with a uniform curvature $\kappa_{C}$), a spatially distributed pressure $P_{E}$ is supplemented to balance the normal component of the force $N_{E}$ acting on the contour of the electrode:

(15) $$P_{E}=2N_{E}\kappa_{C}$$

which ensures a self-equilibrated actuation since the strain actuation forces are always internal forces [4,45]. In this paper, we extend those equivalences to a surface with arbitrary curvatures, where the mean curvatures of spatial distribution are used to describe the profile of the mirror surface. An illustrative diagram of equivalent loads is shown in Figure 3, considering a given point on the mirror surface with principal curvatures $\kappa_{u}$ and $\kappa_{v}$ along orthogonal axes *u* and *v* defined in the sense of Gaussian curvatures, although there will be a coordinate transformation from o${}_{xy}$ to o${}_{uv}$, the Gaussian curvature $\kappa_{G}=\kappa_{u}\kappa_{v}$ and mean curvature $\kappa_{M}=\left(\kappa_{u}+\kappa_{v}\right)/2$ are invariant to the coordinate system. For a small area *dudv*, the supplemented pressure along the *u* and *v* axes are, respectively, $p_{u}=N_{E}\kappa_{u}$ and $p_{v}=N_{E}\kappa_{v}$, with the overall equivalent pressure

(16) $$P_{E}=N_{E}\left(\kappa_{u}+\kappa_{v}\right)=2N_{E}\kappa_{M}$$

**Figure 4:**
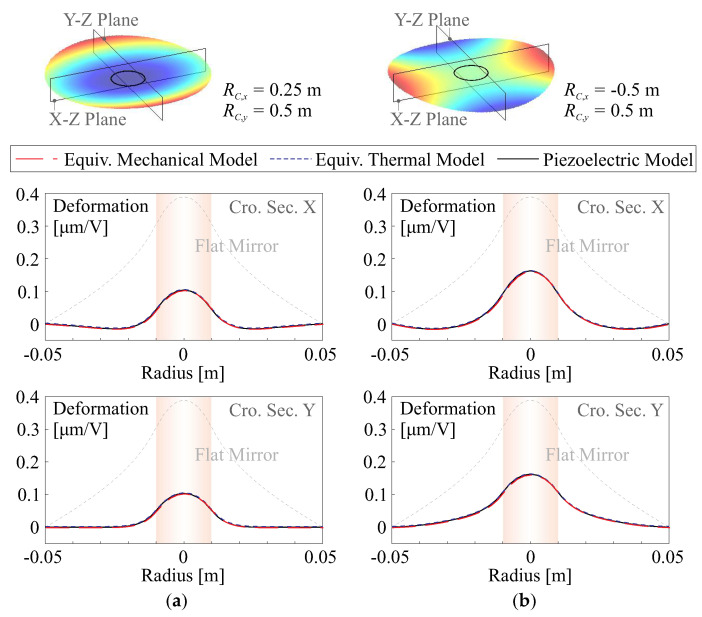
Numerical comparisons of cross-sections of the shape deformation among models of equivalent forces (combining Equations (14) and (16)), the thermal model ($d_{31}V/t_{p}=\Delta \alpha_{T}\Delta T$) and the piezoelectric model; doubly curvature mirrors are unimorphly driven by a central electrode: (**a**) orthogonal curvatures have the same signs, $R_{C,x}=0.25$ m, $R_{C,y}=0.5$ m; (**b**) orthogonal curvatures have different signs, $R_{C,x}$ = −0.5 m, $R_{C,y}=0.5$ m.

Figure 4 shows two numerical examples for verifying Equation (16) of comparing piezoelectric and equivalent loading models. In the tests, the mirrors are moulded as bi-conic shapes, a central circular unimorph strain actuator is used with a diameter of $D_{A}=20$ mm and applied with a unit voltage, and the cross sections of the deformation along orthogonal axes are computed. In more general situations, the piezoelectric actuation can be also modelled using a thermal equivalence, since both piezoelectric actuation in $d_{31}$ mode and the transverse thermal expansion behave identically with $d_{31}vt_{p}=\Delta \alpha_{T}\Delta T$. The numerical comparison is conducted with the Finite Element program, which confirms the good agreement between the equivalent forces, the thermal model, and the piezoelectric model. In addition, Figure 4 also presents the deformation of a flat mirror, indicating that the curvature-induced stiffness significantly reduces the morphing amplitude, which deteriorates the morphing capability.

**Figure 5:**
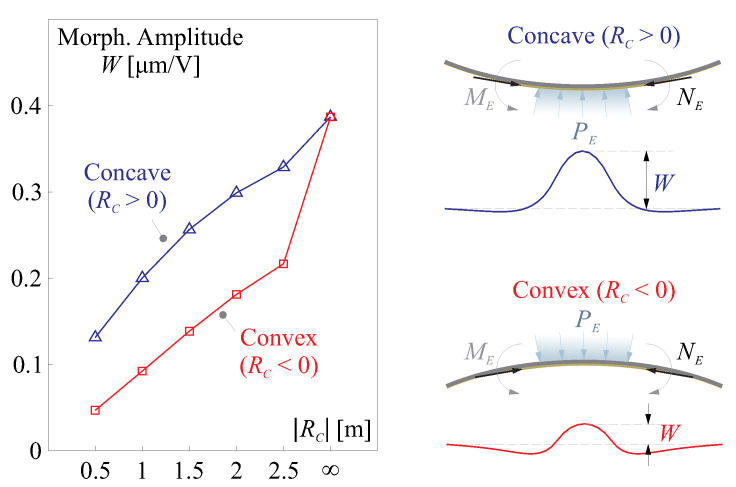
Comparison of the morphing strokes (in μm/V) between concave and convex mirrors with respect to the radius of curvature $R_{C}$, shown on the left side; this depends on the directions of the virtual equivalent pressure $P_{E}$ of Equation (16) and the deformation made by the moment $M_{E}$ of Equation (14) illustrated on the right side.

According to this model of equivalent loads, we can conclude another interesting feature of the morphing behaviour for a composite mirror with dominant curvatures. As shown in Figure 5, the morphing stroke (in μm/V) of a concave mirror is usually larger than that of a convex mirror, in case of a transverse piezoelectric constant of the film $d_{31}<0$ (commonly for the piezoelectric ceramics). This depends on the directions of the virtual equivalent pressure $P_{E}$ of Equation (16) and the deformation made by the moment $M_{E}$ of Equation (14).

Besides the nominal shape (flat or conic) for the optical design, the aberrated shape (the deviation $s_{d}$) is another factor affecting the morphing mechanics if is the amplitude of the abberation is large. The shape deviation can be made by the manufacturing process or released with a complex stress status. Changes on piezoelectric forces also follow Equations (14) and (16); however, they are hard to predict since the irregular $s_{d}$ can be considered to be arbitrarily deformed, in which the uncertainties remain in the magnitudes and directions of those loads. Therefore, statistic studies are conducted to evaluate the influence of shape abberation on the structural mechanics of morphing behaviours.

**Figure 6:**
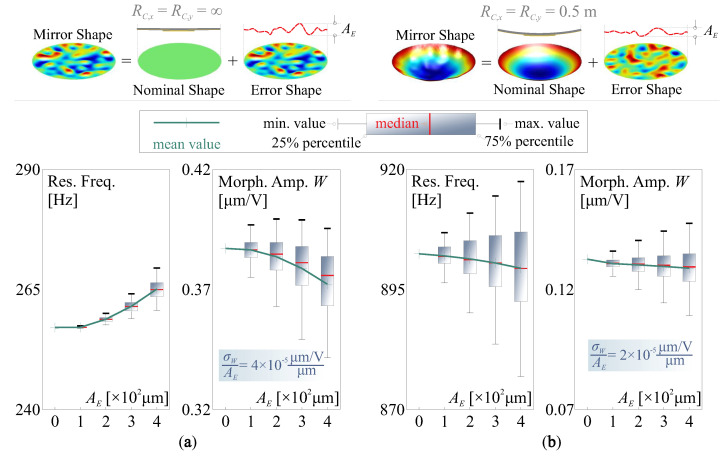
Statistical studies for the effects of the deviated shape on the morphing behaviour and the structural dynamics, the results are presented in the format of a boxplot: (**a**) flat nominal mirror; (**b**) concave nominal mirror $R_{C,x}=R_{C,y}=0.5$ m.

Figure 6 reports statistical analysis for the effects of the deviated shape on the morphing behaviour (actuation strokes, in μm/V) and the structural dynamics (resonant frequencies, in Hz), both flat and conic unimorph mirrors are studied with the identical actuator as used in Figure 4. In these numerical tests, the amplitude of the aberration $A_{E}$ is used as the controlled variable and 100 random samples of $s_{d}$ with arbitrary sets of coefficients of vibration modes are superimposed to the nominal shape for the simulation. The results are presented in the format of a boxplot. Figure 6a shows the stiffening effect of $s_{d}$ for the flat structure of the mirror, the structural rigidity of the mirror is augmented with the increase in $A_{E}$. On the other hand, if the mirror is moulded with dominant curvatures (usually leading to a higher resonant frequency), e.g., the concave mirror in Figure 6b with $R_{C,x}=R_{C,y}=0.5$ m, the initial shape deviation $s_{d}$ weakens the intrinsic stiffness induced by the curvatures, exhibiting an overall softening effect in the sense of statistics. In both cases, the mean values of morphing strokes are slightly reduced in the presence of shape aberration; it can be observed that the uncertainties (characterized by the standard deviation of morphing amplitude $\sigma_{W}$) are proportionally related to $A_{E}$, i.e., $\sigma_{W}/A_{E}$ can be considered as a constant, for a certain nominal shape.

#### 3.2.1. Compensation of Initial Shape Aberration

**Figure 7:**
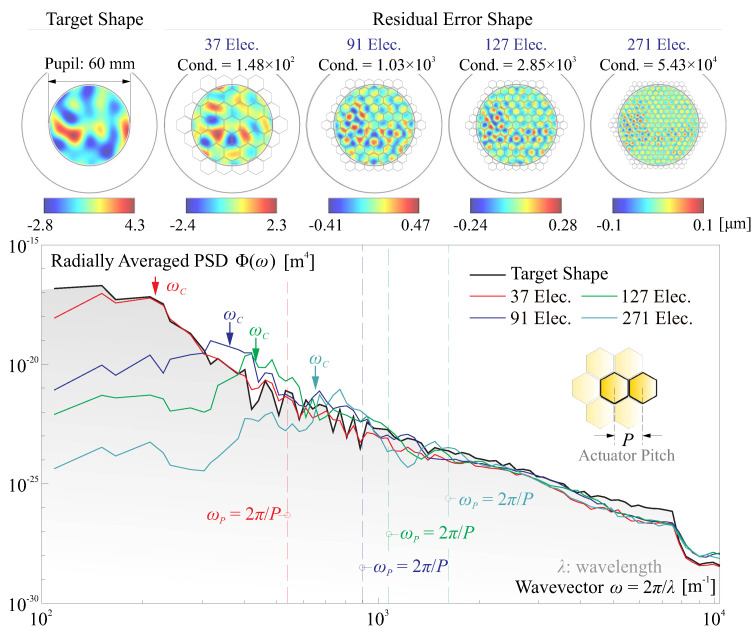
Filtering effect of deformable mirrors: the deformable mirror acts as a low-pass filter in spatial domain, the corner frequency of the PSD for the residual shape increases with the electrode numbers.

If an initial deviated shape exists, the first task of the active optics system will be to correct those aberrations. In this scenario, $s_{d}$ are usually varying in a low bandwidth, and the Least Squares reconstruction of voltage array $v_{t}$ can be used for the quasi-static compensation process. This paper compares both spatial features of the aberrated shape and the actuator patterns, where the radially averaged Power Spectral Density (PSD) is used [46], enabling a one-dimensional measure on the information of spatial frequencies for a two-dimensional surface. Figure 7 presents the computed results for compensating a random deviated shape (target shape), the correction takes places within the pupil $D_{P}$, various patterns of honeycomb configuration with different number of electrodes are tested.

The target shape and the residual error shape after correction are plotted on the top of Figure 7, with an increasing condition number of *J* with the numbers of the actuators. On the bottom of Figure 7, the radially averaged PSDs Φ(ω) are given for those shapes, where ω=2π/λ is the circular spatial frequency, λ is the wavelength describing the characteristic dimension radially of the shape. Those results indicate that the deformable mirror acts as low-pass filter in spatial domain, the corner frequency of the PSD for the residual shape increases with the electrode numbers. In this paper, the frequential components of the PSD can be depicted by the central frequency, defined by the *Rice Formula*:

(17) $$\omega_{C}=\sqrt{\frac{\int_{0}^{\omega_{max}}\omega^{2}\Phi \left(\omega \right)d\omega }{\int_{0}^{\omega_{max}}\Phi \left(\omega \right)d\omega }}$$

The correcting capabilities in spatial frequencies of a certain electrode pattern can be simply quantified by $\omega_{P}=2\pi /P$, where *P* is the actuator pitch; both $\omega_{C}$ and $\omega_{P}$ are marked in Figure 7. Based on the simulation, a rough relationship between those indicative frequencies can concluded by $\omega_{P}/\omega_{C}$ = 2–3, and this indicates that, in this numerical example, a deformable mirror is capable of filtering out those errors with the spatial component at least twice larger than the actuator pitch. The conclusion also implies that the manufacturing error for installing patterned electrodes might always introduce an error that cannot be corrected by the actuator array itself, since the induced aberrations commonly have an influencing area smaller than that of an electrode [11].

To correct the initial shape errors, we see two limitations, first, the amplitude of the deviation $s_{d}$ may not be too large, otherwise huge voltage consumptions might be needed, reducing the input budget for forming $s_{t}$; second, the error components with a high spatial frequency cannot be corrected with a limited number of actuators. Both limitations pose stringent requirements on the error of the mirror profile in both magnitudes and frequential components. Additionally, those also call for possible usage of ultra-high voltage amplifiers in space. According to Figure 7, with a certain actuator array and greater capability of correcting errors with low spatial frequencies, this allows a larger margin of magnitudes of the deviation for simple modes, relaxing the error budgets for the manufacturing and deployment.

**Figure 8:**
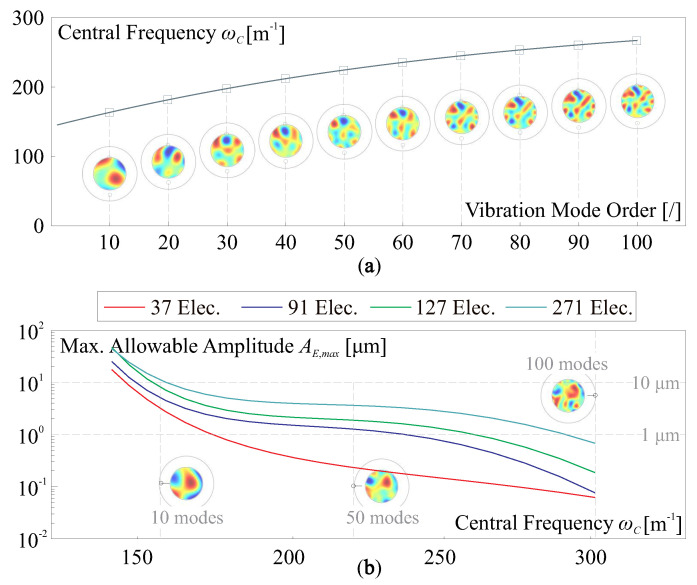
Spatial information of an error shape and the compensation: (**a**) an augmented central frequency $\omega_{C}$ of a shape superimposed with incremental orders of natural modes; (**b**) the maximum allowable $A_{E,max}$ with respect to the central frequency $\omega_{C}$ of the error shape, and various electrode patterns shown in Figure 7 are tested.

The initial deviation and shape deformation can be always decomposed as a set of orthogonal elastic modes within a full mirror structure [20,47], the mode sets are denoted by the matrix Ψ, i.e., within the pupil, $s_{d}=C\Psi z$, where z is the coefficient vector for the modes. The higher orders of vibration modes lead to more complex shapes with high spatial frequencies. Figure 8a shows an augmented central frequency $\omega_{C}$ of a shape superimposed with incremental orders of natural modes, the results are averaged for smoothing with five random samples. In case that a shape error $s_{d}$ is reconstructed with the Least Squares method, the RMS of the residual error $∥\left[I-J{\left(J^{T}J\right)}^{-1}J^{T}\right]s_{d}{∥}_{2}$ is proportional to the amplitude $A_{E}$ of $s_{d}$, and thus the maximum allowable $A_{E,max}$ can be inversely computed with a predefined threshed of the RMS error $\sigma_{W}$. Figure 8b computes $A_{E,max}$ calibrated with $\sigma_{W}=50$ nm, the maximum allowable $A_{E,max}$ is a decreasing function of the central frequency $\omega_{C}$ of the error shape. In this example, various electrode patterns (used in Figure 7, for different *J*) with distinguishable capabilities of reconstructing targets are tested, showing a superior morphing capability for the active mirror with more actuators, to correct an initial deviation with a large amplitude and frequential errors of high orders.

### 3.3. Stress Induced Geometric Stiffness in Composite Mirrors

Another factor altering the system Jacobian *J* of a deformable mirror can be the stress-induced stiffness, which influences the term of ΔK in Equation (6), the stress status of a mirror surface can be changed by two types of sources physically, the first one is the tension or compression forces on the installing boundaries, in the numerical example of this paper, they are acting on the outer rim of the mirror by the supporting frame, possibly due to differences in thermal expansions or mounting stress; the second one is the residual stress that remained within the composite layers generated during the on-ground manufacturing. Those causes usually correspond to different constraints for the structures, e.g., planar membrane forces in the mirror substrate and mismatch strains within layers of the composite. Usually, the former changes only the rigidity of the mirror plate, while the latter might generate bending moments, which deform the mirror, causing an initial error to be corrected, and the remaining stress is still taking the effects of modifying the geometric stiffness. Because those stresses are partially released, we use a factor of β to quantify the stress release in the following discussion, i.e., if β=0, the structure is restrained and the internal stress is contributed to the geometric stiffness, and if β=1, the stress is fully released with a maximum amplitude of the initial error formed.

#### 3.3.1. Change of Morphing Strokes by Edge Loads

We firstly analyse the stress status made by membrane forces from the supporting frames, inspired by the Fourier transform, the forces along the mirror periphery $N_{L}$ (in N/m) might be commonly decomposed with harmonic components of different orders, and the conjugated modal expressions can be written as

(18) $$N_{L}\left(\theta \right)=A_{N}\cos\left(i\theta \right)$$

where θ is the azimuthal coordinate, $A_{N}$ is the amplitude of the harmonic function, and *i* is the order index of the modal component. Note that additional conditions have to be applied on the value of *i*, to ensure a balancing of applied loads, i.e.,

(19) $$\int_{0}^{2\pi }N_{L}\left(\theta \right)\cos\left(\theta \right)=\int_{0}^{2\pi }N_{L}\left(\theta \right)\sin\left(\theta \right)=0$$

**Figure 9:**
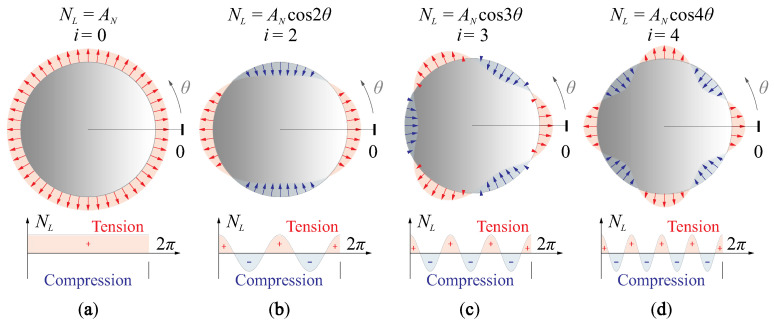
Harmonic loadings along the mirror periphery of different orders: (**a**) i=0; (**b**) i=2; (**c**) i=3; (**d**) i=4.

Figure 9 gives several modal representations of the rim forces with various orders, which will be analysed in this paper.

If the membrane stress is present within the mirror substrate, there will be a special scenario requiring our attention, in case of an overall compressive load inducing a negative geometric stiffness, the mirror (or its structure components) might be buckled with instable deformation when the load reaches a critical level. Of course, we shall avoid this at all the cost for the active mirror in space with environmental uncertainties. A linear buckling analysis for elastic structures consists in solving an eigenvalue problem

(20) $$Kp=\gamma_{B}\Delta Kp$$

in which *K* is the stiffness matrix, ΔK is the geometric stiffness caused by the unit (modal) load, **p** is the buckling modes and $\gamma_{B}$ is the associated amplification factor for the applied unit load; the components of vector **p** are the structure’s DoFs **q**. According to [48], the critical load of buckling $N_{cr}$ for a circular plate can be always proportional to the fundamental structural factor of $D_{B}/R^{2}$ where $D_{B}=Et^{3}/12\left(1-\nu^{2}\right)$ is the flexural rigidity of a plate and R=D/2 is the radius. In the following tests, we use the modal loads illustrated in Figure 9 with an amplitude of $A_{N}=\gamma_{i}D_{B}/R^{2}$ for the load mode *i*, and γ is the loading factor.

**Figure 10:**
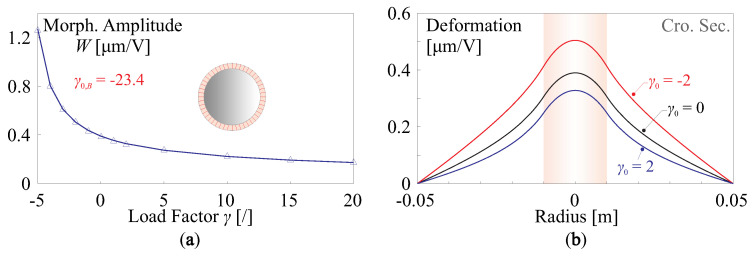
Change on the morphing stroke by the uniform edge loads (illustrated in Figure 9a): (**a**) morphing stroke (in μm/V) with the loading factor γ; (**b**) cross-sections of the deformation for various loading factors, morphing amplitudes with distinct differences can be observed for $\gamma_{0}=$−2, 0, 2.

Figure 10 presents the change of the morphing behaviour of a flat active mirror with respect to uniform edge loads (see Figure 9, i=0), the configuration the piezoelectric actuator follows the numerical examples in Figure 4. The study is conducted with a FE program for the simulation of elastic stability. Figure 10a indicates a tension load increases the rigidity of the structure, resulting in a reduced morphing amplitude, and the actuator stroke can be augmented if a slight compressive load is used. The load factor for buckling is $\gamma_{0,B}=-23.4$, showing possible instability when exciting the structure of the mirror. Note that the value of $\gamma_{B}$ is usually overestimated if the linear mechanics is assumed, it can be much lower in practise. The cross-sections of the deformation are shown in Figure 10b for various loading factors, morphing amplitudes with distinct differences can be observed for $\gamma_{0}=-2,0,2$. It is worth mentioning that the edge load can be also equivalently generated by the expansion or shrinkage of the piezoelectric film driven by the voltage, e.g., consider a fully covered unimorph actuator ($D=D_{A}$), an uniform edge load of $N_{L}=\gamma_{0}D_{B}/R^{2}$ is applied with $\gamma_{0}=-2$ (the same level as computed in Figure 10), according to Equation (14), the equivalent voltage is v$=N_{L}/e_{31}\approx 35$ V, within a common operation range.

**Figure 11:**
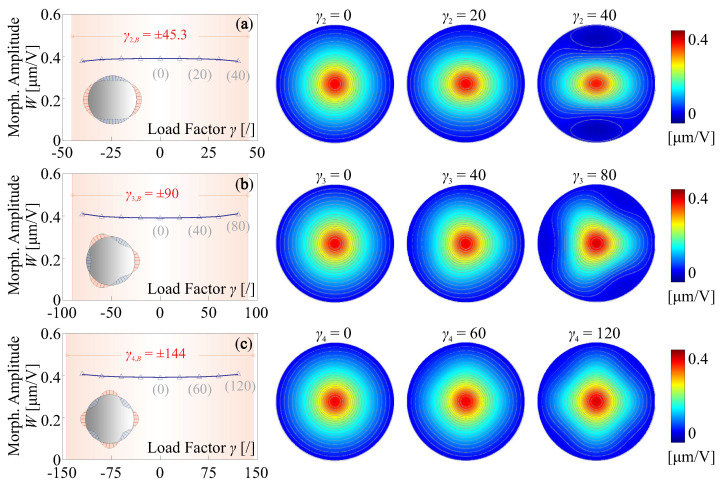
Change on the morphing stroke by edge loads of high modal orders (illustrated in Figure 9b–d), the shape deformations of the active mirror for typical values of $\gamma_{i}$: (**a**) i=2; (**b**) i=3; (**c**) i=4.

Different phenomenon can be found if the edge load has a modal pattern of high orders, e.g., as shown in Figure 9b–d. First, due to an zero-integral for the edge load on the radial direction along the mirror periphery, the buckling load factor $\gamma_{i,B}$ should be expressed in the sense of absolute values, for those (symmetric) loads with high-order modal patterns; second, the absolute value of the buckling factor $|\gamma_{i,B}|$ increases with the order of the load modes; third, the fringe loads have little impacts on the morphing strokes of the unimorph actuator, while larger forming curvatures can be observed along the radial directions of compressive forces on the rim, as indicated in Figure 11.

#### 3.3.2. Release of Internal Stress among Layers

Other sources for stress-induced stiffness can be unbalancing strains among layers of the composite mirror, usually, under a nearly zero-gravity environment of space, the residual stress is partially released with a factor of β quantifying the transformation from the internal stress to the structural deformation, which generates randomly distributed bending moments acting on the mirror surface. The parasitic deformation (considered as a deviated shape $s_{d}$) can be corrected by a designated actuator array (discussed in Section 3.2.1), and the remaining stress slight alters the rigidity of the layered mirror, with the same manner studied in Section 3.3.1. In this section, we focus on the change of the frequential components, from the spatially distributed residual stress among mismatched layers, to the deformed surface of the mirror with the stress released.

**Figure 12:**
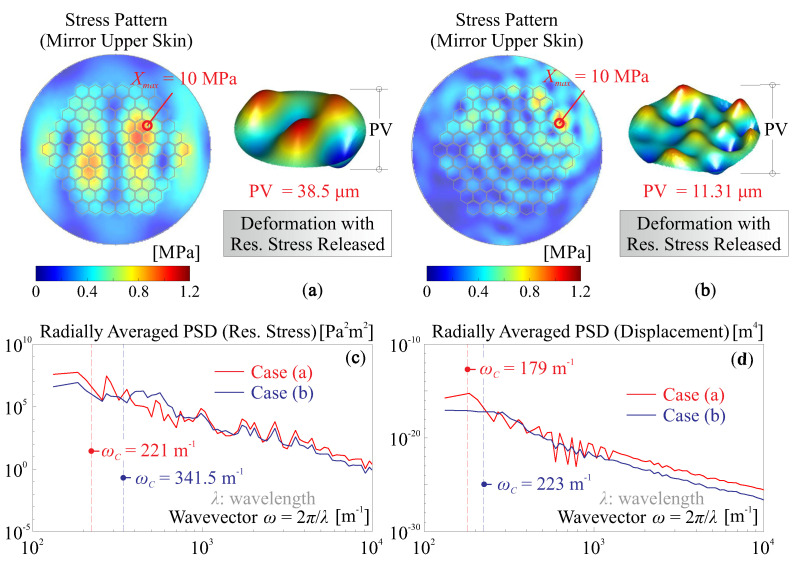
Stressrelease of a layered mirror with modal stress patterns: (**a**) the mirror shape is formed by an initial stress pattern with the first 10 orders of modal stress retained, the maximum value of the upper skin stress is $X_{max}=10$ MPa; (**b**) the same numerical example as case (**a**) except for a retaining order of 50 for the stress pattern is used; (**c**) the radially averaged PSD of the stress on the upper skin of the mirror substrate for case (**a**,**b**); (**d**) the radially averaged PSD of the deformed shape of a full stress release for case (**a**,**b**).

In this numerical study, the simulation analysis involves two steps: first, the modal analysis is conducted for computing modal stress [49] for a layered composite; second, those modal stress patterns are combined with a random set of coefficients, acting as excitation to deform the mirror. Figure 12 shows two examples of the deformation by the stress release, Figure 12a considers a stress pattern of first 10 modes as superimposed, with a maximum value of $X_{max}=10$ MPa on the upper skin of the mirror plate, and if the stress is thoroughly released, the mirror substrate can be deformed accordingly with an amplitude of PV=38.5 μm; another case is if 50 modes are retained for internal stress patterns with the same maximum value of the stress concentration, then the deformation is PV=11.31 μm. The radially averaged PSDs of the upper skin stress and the deformed shape for both cases are presented in Figure 12c–d, respectively, with the central frequencies $\omega_{C}$ indicated. According to those results, we see a complex trade-off relationship among those spatially frequential quantities of the stress and deformation, from a viewpoint of the capability of error correction. If a stress pattern of low-order spatial irregularities is present (e.g., as illustrated in Figure 12a), the free-formed mirror will accordingly have a shape of low-order spatial frequencies, but with a large deforming amplitude, resulting in contradictory quantities on the error shape. In addition, compare Figure 12c,d, we observe that the central frequencies for the PSD of stress pattern are most likely larger than those of the formed shapes.

## 4. Influence on the Jacobian Matrix by Uncertain Factors and Control Strategies

Involving various environmental factors acting on electromechanical behaviour of a deformable mirror for space active optics, the variation of morphing capabilities has been analysed in the sense of mechanics in the previous discussion. This section focuses on the impacts of those factors on the matrix properties of *J* for quasi-static mapping from the voltage to the shape deformation, and the Singular Value Decomposition (SVD) will be used for the design of control strategies and for simple implementation.

### 4.1. Singular Value Decomposition of Perturbed Jacobian

Consider an overdetermined system for the shape control, where the SVD of the nominal *J* is

(21) $$J=U_{R}\Sigma V^{T}=\sum_{i=1}^{n}\sigma_{i}u_{i}v_{i}^{T}$$

where $U_{R}$ is the range space (with columns of $u_{i}$) of the matrix *J*, and usually *J* and *U* have the same dimensions; Σ is a diagonal matrix with the terms of singular values $\sigma_{i}$ in a descending sequence; *V* is the input unitary matrix with columns of $v_{i}$. The physical interpretations of $U_{R}$, *V*, and Σ for a voltage-driven deformable mirror are possible sets of formed shapes projecting on the sensor ($u_{i}$), and corresponding voltage maps for those formed shapes ($v_{i}$) with associated weight factors ($\sigma_{i}$), respectively.

The change of *J* for Equation (6) is expressed in an additive uncertain matrix of ΔJ, here the SVD of J+ΔJ can be written as $J+\Delta J=U_{1R}\Sigma_{1}V_{1}^{T}$, and another updating algorithm for the SVD of an overdetermined matrix can be found in [50]. Consider a small variation of the Jacobian matrix, i.e., ∥ΔJ∥≪∥J∥, the perturbed Jacobian can be approximated by $J+\Delta J≃U_{R}\left(\Sigma +\Delta \Sigma \right)V^{T}$, note the matrix ΔΣ does not have to be diagonal, and possible off-diagonal terms show the coupling among singular modes. Additionally, it is interesting to observe that ${∥\Sigma +\Delta \Sigma ∥}_{F}={∥\Sigma_{1}∥}_{F}$.

**Figure 13:**
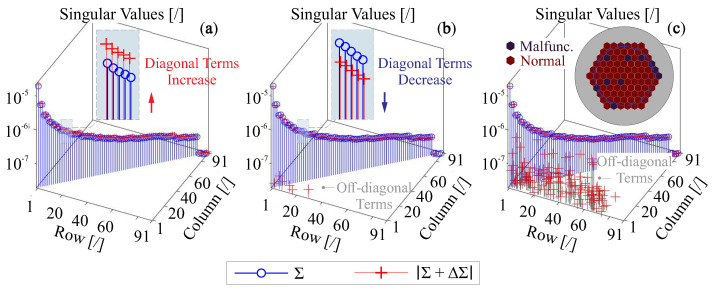
Illustration on the matrix entries of Σ+ΔΣ for various scenarios, the magnitudes are presented, and a deformable mirror with 91 unimorph actuators (see Figure 7) is used: (**a**) a uniform temperature increase of ΔT=30°C is applied; (**b**) remaining residual stress among composite layers is externally applied with the same pattern as shown in Figure 12b with a random edge tension load; (**c**) 10 actuators are malfunctioned, and the damaged actuators are indicated.

Figure 13 gives several illustrative plots on the matrix entries of Σ+ΔΣ for various scenarios of space environment, the magnitudes (absolute values) are presented, and a deformable mirror with 91 unimorph actuators (see Figure 7) is used for the simulation. The impact of the temperature change is presented in Figure 13a, with a uniform temperature increase of ΔT=30°C, according to Equation (6), the scaling factor κ can be used to describe the change of the actuator stroke for each channel, where ΔΣ=(κ−1)Σ, therefore, the variation occurs only on the diagonal terms.

Figure 13b shows the change of singular values for a complex stress status, two kinds of stresses are superimposed to modify the geometric stiffness of the structure, the remaining residual stress among composite layer is externally applied with the same pattern as shown in Figure 12b, and the mirror is also tensioned with a random edge load. Based on the results, the diagonal terms are slightly reduced due to the structural rigidities by external loads. In addition, off-diagonal terms appear because the nonuniform stress induces internal stiffness of a random spatial pattern; although those terms are relatively small compared to the diagonal entries, and they are staying in a region of low order singular modes, which might dominantly influence the mode reconstruction. Figure 13c gives an example of the change of the matrix *B* for Equation (6), which considers that some actuators are malfunctioned. In general, the diagonal matrix *B* means the independent change on each actuator, they can be actuator failure, e.g., the breakdown of dielectric material, or partially deteriorated, e.g., the aging of the piezoelectric film, the morphing stroke attenuation by the dehumidified space environment, noises and biases of voltage amplifiers. Currently, there is no clear theoretical formulation for the distribution of where the damage occurs, in a practical sense, those damaged actuators tend to be clustered rather than being uniformly distributed (especially for the electrical breakdown). In this example, we assume 10 randomly selected actuators are damaged, and the results show that the matrix *J* can be significantly disturbed with a huge amount of coupling noises, indicating the range space $U_{R}$ might be reduced, i.e., some vectors of $u_{i}$ are moved to the null space and the sequences might also be changed, other random numerical examples show a similar trend. Usually, a robust design of the electrode pattern for the deformable mirror allows a maximum number of 5% actuators to be in a failure condition [51].

### 4.2. Open Loop Control and Iterative Control

The shape control using the strategies of inverting *J* can be also evaluated in a sense of eliminating terms for the range space in the target shape. Consider a Least Squares correction, $s_{e}=\left(I-JJ^{+}\right)s_{t}$, the operator matrix between $s_{e}$ and $s_{t}$ can be also written as $I-JJ^{+}=I-U_{R}U_{R}^{T}$. If the system is perturbed with an update of the range space from $U_{R}$ to $U_{1R}$, the transform can be understood as a change of basis, and the lost parts from the original space can be written as

(22) $$U_{e}=\left(I-U_{1R}U_{1R}^{T}\right)U_{R}$$

The columns of $U_{e}$, denoted by $u_{e,i}$, are the residual errors of reconstructing $u_{i}$, the columns of $U_{R}$; the overall RMS error for this can be quantified by $∥U_{e}{∥}_{F}$, which is a measure of the loss of the column space by changing from *J* to J+ΔJ. In case that $∥U_{e}{∥}_{F}≃0$, the set of formable shapes for the active mirror will remain unchanged to the variation of *J*, and those shapes can be also generated once a proper inversion of the system can be solved even if ΔJ exists. The values of $∥U_{e}{∥}_{F}$ for cases in Figure 13 are 0, $8.4\times 10^{-3}$ and 3.16, respectively. The difference for those values indicates that the range space of the Jacobian remains if there are scalings on the morphing strokes for the actuators, while the malfunctioning of actuators might cause irrecoverable loss of morphing modes. Note that no weighting is considered for computing $∥U_{e}{∥}_{F}$ for forming the range space of *J*, i.e., the voltage consumption might be unrealistically huge when solving Equation (22). If weighting factors of normalized singular values $\sigma_{i}/\sigma_{max}$ are multiplied, we can compute $∥U_{e}\cdot diag\left(\sigma_{i}/\sigma_{max}\right){∥}_{F}$, with values of 0, $5.93\times 10^{-5}$, and $2.25\times 10^{-2}$, respectively, and this eliminates the effect of unnecessary control efforts for high order singular modes.

In general, the entries of ΔJ are unknown, although those can be analytically approximated for solving the inverse problem. If we assume the information of ΔJ is unknown or its entries are statistically white, the voltage vector v can be computed by Equation (3) or Equation (4) with a nominal *J*, which is considered to be known for the control process. On the other hand, the correcting shape should also include the excited deviation $s_{d}$ associated with environmental factors, i.e., the overall target for the compensation is $s_{t}-s_{d}$. In this case, the Least Squares algorithm leads to a reconstructed shape of $s_{r}=\left(J+\Delta J\right)J^{+}\left(s_{t}-s_{d}\right)$, and the error shape can be

(23) $$s_{e}=\left[I-\left(J+\Delta J\right)J^{+}\right]\left(s_{t}-s_{d}\right)$$

showing that both ΔJ and $s_{d}$ might deteriorate the shape control.

Another control strategy is to make the correction iteratively by discrete steps, and the matrix inversion of Damped Least Squares is employed. The incremental voltage vector $v_{k}-v_{k-1}$ is computed (*k* is the step index) to correct the error shape of the $k^{th}$ step, i.e.,

(24) $$v_{k}-v_{k-1}={\left(J^{T}J+\alpha^{2}I\right)}^{-1}J^{T}\left[s_{t}-s_{d}-\left(J+\Delta J\right)v_{k-1}\right]$$

Note that the computation of the DLS inverse operator is based on the nominal *J*. According to Equation (24), we can obtain the corresponding recurrence equation

(25) $$v_{k}={\left(J^{T}J+\alpha^{2}I\right)}^{-1}\left(\alpha^{2}I-J^{T}\Delta J\right)v_{k-1}+{\left(J^{T}J+\alpha^{2}I\right)}^{-1}J^{T}\left(s_{t}-s_{d}\right)$$

to examine the convergent behaviour of the iterative steps.

To examine the stability (convergency to a steady state) of the iteration, we may define the recurrence matrix $P={\left(J^{T}J+\alpha^{2}I\right)}^{-1}\left(\alpha^{2}I-J^{T}\Delta J\right)$ in such a way that the convergency depends on the criteria ${∥P∥}_{2}\leq 1$. In particular, if ${∥\Delta J∥}_{F}=0$, the condition of ${∥P∥}_{2}=\alpha^{2}/\left(\sigma_{min}^{2}+\alpha^{2}\right)\leq 1$ can be always guaranteed, where $\sigma_{min}$ is the smallest value of the retained sequence $\sigma_{i}$. A more general expression for the norm of the recurrence matrix is

(26) $${∥P∥}_{2}={∥{\left(\alpha^{2}I+\Sigma^{2}\right)}^{-1}\left(\alpha^{2}I-\Sigma \Delta \Sigma \right)∥}_{2}$$

where the disturbing term ΔΣ can be computed by $\Delta \Sigma =U_{R}^{T}J_{1}V-\Sigma$. The value of ${∥P∥}_{2}$ controls the iterative efficiency, a smaller value means fast convergency. Table 1 reports the computed results of ${∥P∥}_{2}$ for various situations illustrated in Figure 13, which decreases with a reduced damping factor $\alpha^{2}$, an augmented morphing capability (e.g., caused by a temperature increase or compressive edge loads) performs a successive overrelaxation factor in the iterative process. It is worth mentioning that, if there are a certain number of actuators in a failure condition, the voltage computation might go to a divergent status, and the simulated study shows a good consistently (i.e., malfunction ratio: 5/91≃5.5%) with the threshold proposed in [51].

If the convergency of the control loop can be ensured, then the vector of final voltage $v_{F}$ for the steady-state shape can be computed by assuming $v_{k}≃v_{k-1}$, we can substitute this into Equation (25), with a solution of $v={\left(I-P\right)}^{-1}{\left(J^{T}J+\alpha^{2}I\right)}^{-1}J^{T}\left(s_{t}-s_{d}\right)$. Therefore, the error shape for the iterative control is

(27) $$s_{e}=\left\{I-\left(J+\Delta J\right){\left[J^{T}\left(J+\Delta J\right)\right]}^{-1}J^{T}\right\}\left(s_{t}-s_{d}\right)$$

**Figure 14:**
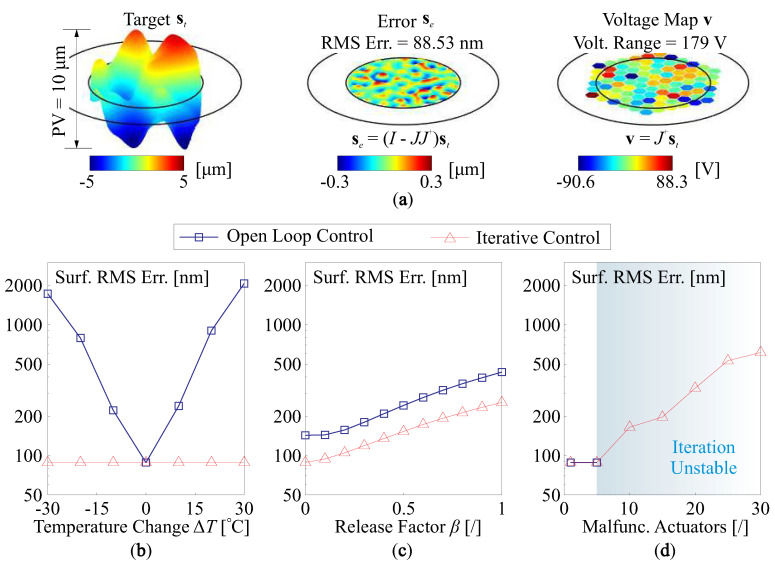
Open loop and iterative control methods: (**a**) the open loop shape correction with $s_{d}=0$, the target shape is designated with PV=10 μm; (**b**) the compensation of $s_{t}-s_{d}$ made by the changes of thermal environment in space; (**c**) the compensation of the error shape released by the nonuniform remaining stresses among layers of the composite mirror, the mirror is also subjected to a random edge tension; (**d**) the shape forming of $s_{t}$ when some of actuators are malfunctioned, the iterative method does not work if more than 5 actuators are broken down.

If ${∥\Delta J∥}_{F}=0$, this leads to a Least Squares solution. Note that this can be significantly different to the open-loop solution by Equation (23). Figure 14a shows the open-loop control process for a target shape $s_{t}$, the required forming amplitude within a clear pupil is PV=10 μm; and Figure 14b–d compare the residual error after control between the open-loop and iterative methods for the shape $s_{t}-s_{d}$, in addition, the input voltage range is limited with a range of −300–400 V. In Figure 14b, the deviated shape $s_{d}$ is caused by the change of thermal environment, and the mirror is excited by the variation of thermal and poling strains. The open-loop control produces a huge error in the residual shape because the computation of v cannot involve the change of the Jacobian ΔJ, and this error can be thoroughly eliminated by the iterative method, with a result of the RMS of residual errors close to that of Figure 14a, the deviated shape $s_{d}$ made by this kind of uniform strain commonly generates a shape error of low spatial frequencies. For summary, the operating temperature of a unimorph DM should be limited due to two main effects: (1) a large temperature change can lead to deformation caused by a mismatch between the substrate and the active film, altering the poling strain; (2) the piezoelectric coefficient is temperature-sensitive; while these effects can be mitigated by a feedback control loop, as shown in Figure 14b, the limiting factor becomes the actuator saturation.

Figure 14c shows the RMS of residual errors with an increase in release factors, the free-formed shape by random stress among the layers and edge loads might exhibit a pattern of high orders, which poses difficulties on using both the open-loop and iterative control methods. The iterative shaping process shows better performances at the steady state because the equivalent inverting process of Equation (24) considers the update matrix J+ΔJ rather than *J*. Despite an elaborate control design, a fine manufacturing process would still be required, especially for the attenuation of the residual stresses during layering, where proper annealing might be tested. Figure 14d presents those comparisons when some of the actuators are malfunctioned (assuming $s_{d}=0$), according to Table 1, the iteration will not be convergent if more than 5 actuators are broken down, and in this case, a periodical open-loop control should be used, although better performances are not expected.

## 5. Conclusions

This paper discusses the change of morphing capabilities for a unimorph piezoelectric deformable mirror impacted by environmental factors, which works in space for the applications of active optics. Various aspects of disturbing sources are considered for both perturbed shape and the Jacobian matrix: first, the nonlinear ferroelectric behaviour of the active film under complex thermal and mechanical conditions is analysed using a phenomenological model based on Landau and Devonshire’s theory, the shape change with a large temperature range is simulated, with both thermal expansion and change of poling strains taken into account. Second, the intrinsic rigidities of the mirror structure induced by the principle curvatures are simulated numerically and verified by a model of decomposing the strain actuation into equivalent loads, this can be observed if a preconfigured shape is defined. Additionally, the statistics of morphing strokes and structural dynamics with random initial shape errors are computed. The deformable mirrors with various actuator arrays can be also regarded as spatial filters, and frequential quantities relating to the target and residual shapes are studied. Third, the stress-induced geometric stiffness is considered with two scenarios, where the first scenario considers that the structure stiffness is altered by edge loads generated by the incompatibilities with the supporting boundaries, and the second scenario studies the modified rigidity introduced by the residual stresses within layers of the composite mirror. Those variations are regarded as uncertainties, and this paper also proposes a method of analysing the perturbed Jacobian matrix by the Singular Value Decomposition (SVD). Finally, the strategies of open-loop reconstruction and iterative control based on the Damped Least Squares method are studied, with various environmental factors applied for the numerical verification.

## Figures and Tables

**Table 1:** ${∥P∥}_{2}$ computed by Equation (26), the values the damping factors are $\alpha =\sigma_{min}$ and $\alpha =3\sigma_{min}$, respectively, where $\sigma_{min}$ is the minimum singular value of *J*. Various factors pertubating *J* are considered for some scenarios: (1) a temperature change with a range of −20–30°C; (2) a set of actuators are malfunctioned; (3) a complex stress status of Figure 12b with a random edge tension is applied.

Temperature Change			Actuators Failure		
ΔT [°C]	$\alpha =\sigma_{\min}$	$\alpha =3\sigma_{\min}$	**Malfunctioning Number [/]**	$\alpha =\sigma_{\min}$	$\alpha =3\sigma_{\min}$
−20	0.521	0.904	0	0.500	0.900
−10	0.511	0.902	5	0.500	0.900
0	0.500	0.900	>5	>1	
10	0.489	0.898	Complex Stress Status		
20	0.477	0.895	Residual Stress in Layers	$\alpha =\sigma_{min}$	$\alpha =3\sigma_{min}$
30	0.465	0.893	with Random Edge Tension	0.526	0.905

## Data Availability

Not applicable.

## Abbreviations

The following abbreviations are used in this manuscript:

AO	Adaptive Optics or Active Optics
CTE	Coefficient of Thermal Expansion
DM	Deformable Mirror
DLS	Damped Least Squares
DoF	Degree-of-Freedom
FoV	Field-of-View
FE	Finite Element
GS	Guide Star
LS	Least Squares
PMN-PT	Lead Magnesium Niobate—Lead Titanate
PSD	Power Spectral Density
PV	Peak to Valley
PVDF-TrFE	PolyVinylidene Fluoride-co-TriFluoroEthylene
PZT	Lead Zirconate Titanate
SLM	Spatial Light Modulator
SR	Strehl Ratio
SVD	Singular Value Decomposition
RMS	Root Mean Square

## References

[1] Trumper I., Hallibert P., Arenberg J.W., Kunieda H., Guyon O., Stahl H.P., Kim D.W. (2018). Optics Technology for Large-aperture Space Telescopes: From Fabrication to Final Acceptance Tests. Adv. Opt. Photonics.

[2] Chen S., Huang H. (2010). Optimum Design of a Space Frame and Its Application in Satellite Structure. J. Spacecr. Rocket..

[3] Meguro A., Shintate K., Usui M., Tsujihata A. (2009). In-orbit Deployment Characteristics of Large Deployable Antenna Reflector Onboard Engineering Test Satellite VIII. Acta Astronaut..

[4] Wang K., Yu Y., Preumont A. (2023). Wavefront Control Strategies for Large Active Thin Shell Primaries with Unimorph Actuators. Actuators.

[5] Roddier F. (1999). Adaptive Optics in Astronomy.

[6] Tyson R.K. (2011). Principle of Adaptive Optics.

[7] Sinquin J.C., Lurçon J.M., Guillemard C. Deformable Mirror Technologies for Astronomy at CILAS. Proceedings of the SPIE Astronomical Telescopes + Instrumentation.

[8] Wlodarczyk K.L., Bryce E., Schwartz N., Strachan M., Hutson D., Maier R.R., Atkinson D., Beard S., Baillie T., Parr-Burman P. (2014). Scalable Stacked Array Piezoelectric Deformable Mirror for Astronomy And Laser Processing Applications. Rev. Sci. Instrum..

[9] Stroebele S., Vernet E., Brinkmann M., Jakob G., Lilley P., Casali M., Madec P.Y., Kasper M. Deformable Mirrors Development Program at ESO. Proceedings of the SPIE Astronomical Telescopes + Instrumentation.

[10] Rausch P., Verpoort S., Wittrock U. (2015). Unimorph Deformable Mirror for Space Telescopes: Design and Manufacturing. Opt. Express.

[11] Alaluf D., Bastaits R., Wang K., Horodinca M., Martic G., Mokrani B., Preumont A. (2018). Unimorph Mirror for Adaptive Optics in Space Telescopes. Appl. Opt..

[12] Wang K., Godfroid T., Robert D., Preumont A. (2020). Electrostrictive PVDF-TrFE Thin Film Actuators for the Control of Adaptive Thin Shell Reflectors. Actuators.

[13] Wang K., Godfroid T., Robert D., Preumont A. (2021). Adaptive Shell Spherical Reflector Actuated with PVDF-TrFE Thin Film Strain Actuators. Actuators.

[14] Cousty R., Antonini T., Aubry M., Krol H.T., Moreau A. Monomorph Deformable Mirrors: From Ground-Based Facilities to Space Telescopes. Proceedings of the SPIE International Conference on Space Optics—ICSO 2016.

[15] Wang K., Alaluf D., Preumont A. (2019). Thermal Balance and Active Damping of a Piezoelectric Deformable Mirror for Adaptive Optics. Actuators.

[16] Uchino K. (2020). Electrothermal Phenomena in Ferroelectrics. Actuators.

[17] Li F., Xu Z., Wei X., Yao X. (2009). Determination of Temperature Dependence of Piezoelectric Coefficients Matrix of Lead Zirconate Titanate Ceramics by Quasi-static and Resonance Method. J. Phys. Appl. Phys..

[18] Zhou D., Kamlah M., Munz D. (2005). Effects of Uniaxial Prestress on The Ferroelectric Hysteretic Response of Soft PZT. J. Eur. Ceram. Soc..

[19] Zhang Q.M., Zhao J. (1999). Electromechanical Properties of Lead Zirconate Titanate Piezoceramics under the Influence of Mechanical Stresses. IEEE Trans. Ultrason. Ferroelectr. Freq. Control..

[20] Preumont A. (2013). Twelve Lectures on Structural Dynamics.

[21] Bastaits R., Alaluf D., Belloni E., Rodrigues G., Preumont A. (2014). Segmented Bimorph Mirrors for Adaptive Optics: Morphing Strategy. Appl. Opt..

[22] Wang K., Alaluf D., Mokrani B., Preumont A. (2018). Control-structure Interaction in Piezoelectric Deformable Mirrors for Adaptive Optics. Smart Struct. Syst..

[23] Lai G., Yatagai T. (1991). Generalized Phase-shifting Interferometry. J. Opt. Soc. Am. A.

[24] Kinnstaetter K., Lohmann A.W., Schwider J., Streibl N. (1988). Accuracy of Phase Shifting Interferometry. Appl. Opt..

[25] Joannes L., Dubois F., Legros J.C. (2003). Phase-Shifting Schlieren: High-Resolution Quantitative Schlieren that Uses the Phase-Shifting Technique Principle. Appl. Opt..

[26] Primot J. (2003). Theoretical Description of Shack–Hartmann Wave-front Sensor. Opt. Commun..

[27] Poyneer L.A. (2003). Scene-based Shack-Hartmann Wave-front Sensing: Analysis and Simulation. Appl. Opt..

[28] Platt B.C., Shack R. (2001). History and Principles of Shack-Hartmann Wavefront Sensing. J. Refract. Surg..

[29] Fienup J.R. (1978). Reconstruction of an Object from the Modulus of Its Fourier Transform. Opt. Lett..

[30] Wu Y., Sharma M.K., Veeraraghavan A. (2019). WISH: Wavefront Imaging Sensor with High Resolution. Light. Sci. Appl..

[31] Buss S.R. (2004). Introduction to Inverse Kinematics with Jacobian Transpose, Pseudoinverse and Damped Least Squares Methods. IEEE J. Robot. Autom..

[32] Damjanovic D. (1998). Ferroelectric, Dielectric and Piezoelectric Properties of Ferroelectric Thin Films and Ceramics. Rep. Prog. Phys..

[33] Chandra P., Littlewood P.B. (2007). A Landau Primer for Ferroelectrics. Physics of Ferroelectrics: A Modern Perspective.

[34] Wang Q.M., Zhang T., Chen Q., Du X.H. (2003). Effect of DC Bias Field on the Complex Materials Coefficients of Piezoelectric Resonators. Sens. Actuators Phys..

[35] Masys A.J., Ren W., Yang G., Mukherjee B.K. (2003). Piezoelectric Strain in Lead Zirconate Titante Ceramics as A Function of Electric Field, Frequency, and DC Bias. J. Appl. Phys..

[36] Yang G., Liu S.F., Ren W., Mukherjee B.K. (2001). Effects of Uniaxial Stress on the Piezoelectric, Dielectric, and Mechanical Properties of Lead Zirconate Titanate Piezoceramics. Ferroelectrics.

[37] Blaurock C., Mcginnis M., Kim K., Mosier G.E. Structural-Thermal-Optical Performance (STOP) Sensitivity Analysis for the James Webb Space Telescope. Proceedings of the SPIE Optics and Photonics 2005.

[38] Nielsen C.J.G., Tian D., Wang K., Preumont A. (2022). Adaptive Deployable Thin Spherical Shell Reflectors. Actuators.

[39] Mao Y., Jiang B., Du J., Yin J. The Simulation Study of Thermal Design Method for Space Optical Window. Proceedings of the 2nd International Forum of Young Scientists on Advanced Optical Manufacturing (AOMTA and YSAOM 2022).

[40] Yang H., Shen F., Zhang Z., Chen L., Wu Q. Thermal Design and Verification of the Large-temperature-difference Space Optical System. Proceedings of the 2017 IEEE International Conference on Mechatronics and Automation (ICMA).

[41] Villalba V., Kuiper H., Gill E. (2020). Review on Thermal and Mechanical Challenges in the Development of Deployable Space Optics. J. Astron. Telesc. Instrum. Syst..

[42] Wang L., Ast D.G., Bhargava P., Zehnder A.T. (2003). Curved Silicon Electronics. MRS Online Proc. Libr. (OPL).

[43] Suzuki S., Matsumoto Y. (2008). Lithography with UV-LED Array for Curved Surface Structure. Microsyst. Technol..

[44] Preumont A. (2018). Vibration Control of Active Structures, an Introduction.

[45] Wang K., Alaluf D., Rodrigues G., Preumont A. (2021). Precision Shape Control of Ultra Thin Shells with Strain Actuators. J. Appl. Comput. Mech..

[46] Persson B.N., Albohr O., Tartaglino U., Volokitin A.I., Tosatti E. (2004). On the Nature of Surface Roughness with Application to Contact Mechanics, Sealing, Rubber Friction and Adhesion. J. Phys. Condens. Matter.

[47] Wang H., Zhang M., Zuo Y., Zheng X. (2019). Research on Elastic Modes of Circular Deformable Mirror for Adaptive Optics and Active Optics Corrections. Opt. Express.

[48] Timoshenko S.P., Gere J.M. (2009). Theory of Elastic Stability.

[49] Preumont A. (1994). Random Vibration and Spectral Analysis.

[50] Peña J.M., Sauer T. (2019). SVD Update Methods for Large Matrices and Applications. Linear Algebra Its Appl..

[51] Madec P.Y. Overview of Deformable Mirror Technologies for Adaptive Optics and Astronomy. Proceedings of the SPIE Astronomical Telescopes + Instrumentation.

